# Isomerism as a Design Tool: Tuning the Energy–Safety Balance of a Fused Pyrazolo-Triazine N-Oxide Energetic Material

**DOI:** 10.3390/molecules31183203

**Published:** 2026-09-11

**Authors:** Haohuang Wang, Ziwu Cai, Tianyu Jiang, Yuteng Cao, Wenquan Zhang

**Affiliations:** 1 Institute of Chemical Materials, China Academy of Engineering Physics, Mianyang 621999, China; wanghaohuang24@gscaep.ac.cn (H.W.); jiangtianyu21@gscaep.ac.cn (T.J.); caoyuteng18@gscaep.ac.cn (Y.C.); 2 Extreme-Environment-Resistant Materials Key Laboratory of Sichuan Province, Sichuan University of Science & Engineering, Zigong 643000, China; caiziwu25@163.com

**Keywords:** energetic materials, isomerism, N-oxide, synthesis, detonation performance

## Abstract

Addressing the inherent trade-off between energy content and safety remains a long-standing priority in the field of energetic materials. In this work, we employed a straightforward isomer design strategy, synthesizing a structural isomer of 4-amino-7-methyl-5-nitro-7H-pyrazolo[3,4-d][1,2,3]triazine-2-oxide (MPTO), namely 4-methylamino-5-nitro-7H-pyrazolo[3,4-d][1,2,3]triazine-2-oxide (1). Despite involving only a subtle difference in the connectivity of substituents, compound **1** exhibits a dramatically distinct crystal packing mode compared to MPTO. This structural variation directly translates into a remarkable performance enhancement: compound **1** not only delivers superior detonation velocity (8193 m·s^−1^) and detonation pressure (25.6 GPa) relative to the parent compound, but also shows substantially reduced mechanical sensitivities (impact sensitivity > 20 J, friction sensitivity > 360 N), while maintaining favorable thermal stability (296 °C). This study not only provides a promising candidate energetic molecule with well-balanced overall performance, but also highlights, from a crystal engineering perspective, the significant potential of isomer design in fine-tuning the properties of fused-ring energetic materials.

## 1. Introduction

Resolving the inherent trade-off between energy density and safety margin represents a central challenge in the field of energetic materials [1]. Fused nitrogen-rich heterocycles have emerged as promising frameworks due to their planar molecular structures, extensive hydrogen-bonding networks, and close crystal packing modes, which collectively offer the potential to mitigate mechanical sensitivity while maintaining high energy output [2,3,4]. Nevertheless, the rational design of fused-ring systems that simultaneously achieve superior detonation performance and low sensitivity remains a formidable task, largely because our understanding of how subtle structural variations influence intermolecular interactions and crystal packing architectures is still limited.

**Figure 1:**
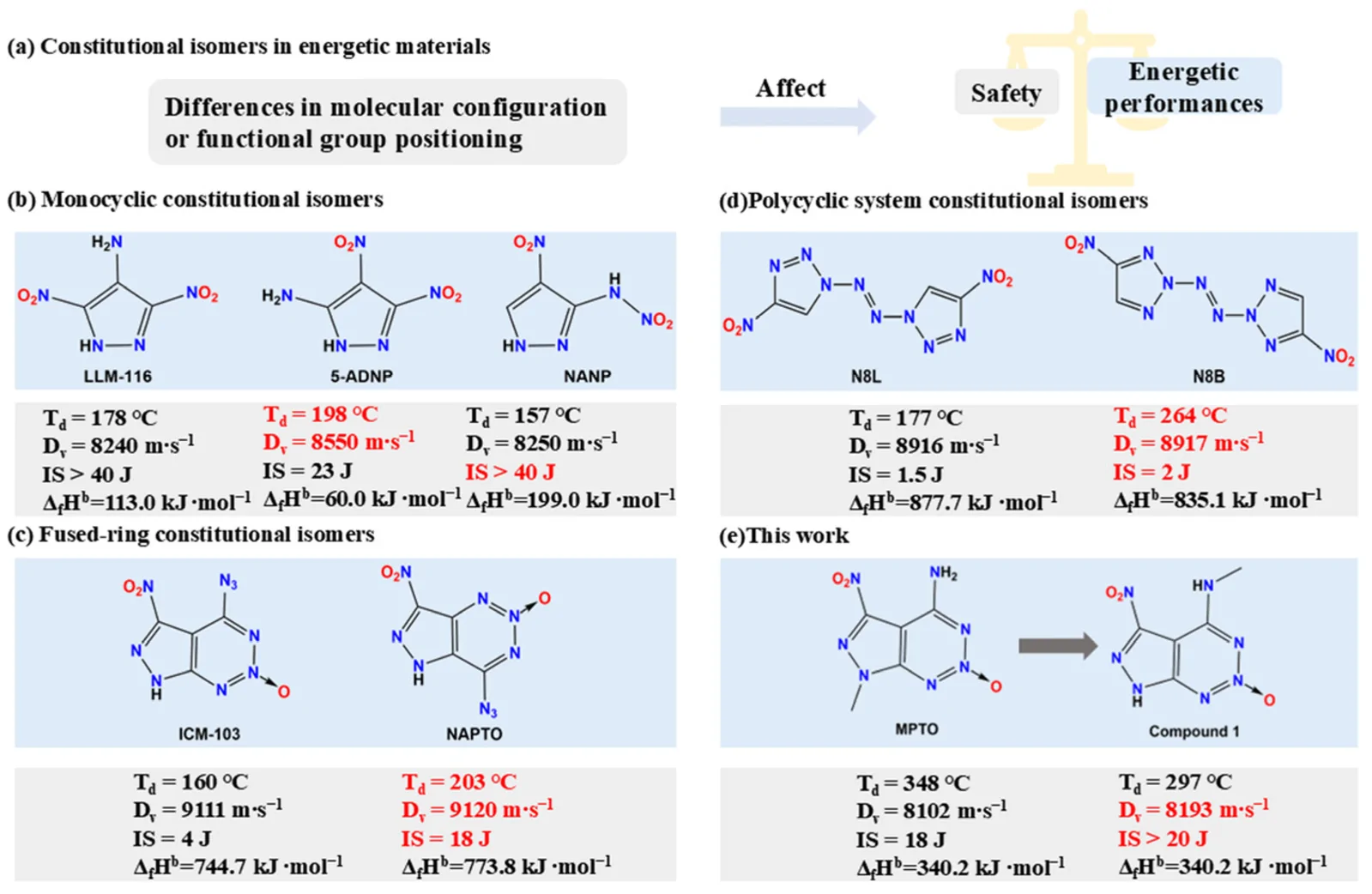
Constitutional isomers in energetic materials.

Isomer design provides an elegant strategy to address this challenge, covering positional isomerism and stereoisomerism [5]. Isomerism has recently attracted increasing attention as a subtle structural regulation strategy for energetic materials. Unlike conventional approaches involving the introduction of new energetic groups or modification of molecular skeletons, isomeric regulation provides an opportunity to tune physicochemical properties while maintaining the same molecular composition. Previous studies have demonstrated that positional isomerism can influence crystal packing, intermolecular interactions, thermal stability, and sensitivity of energetic compounds [6,7,8,9,10,11,12,13,14,15,16,17]. Despite possessing identical molecular formulas and highly similar molecular frameworks, isomers can exhibit significant differences in their physicochemical properties. By relocating a substituent or atom to a different position on a fixed molecular skeleton without altering the molecular formula, one can modulate crystal packing and intermolecular contacts, thereby tuning key parameters such as density, decomposition temperature [18], and sensitivity [19] (Figure 1). Several reported systems support this strategy. For instance, Ma et al. explored 3-nitramine-4-nitropyrazole (NANP) and observed its distinctly different physical and energy properties from its isomers 4-amino-3,5-dinitro-1H-pyrazole (LLM-116) and 5-amino-3,4-dinitropyrazole (5-ADNP) [20,21]. Our group reported a group of isomers with fused-ring structures: 6-nitro-7-azido-pyrazol[3,4-d][1,2,3]triazine-2-oxide (ICM-103) and 4-nitro-7-azido-pyrazol[3,4-d][1,2,3] triazine-2-oxide (NAPTO) [22,23,24,25]. Only the nitro and azido groups occupy different positions. This positional variation alters hydrogen bonding and layered packing, leading to distinct differences in decomposition temperature and sensitivity. In addition, its nitro-substituted derivative was synthesized for comparison (4-amino-5-nitro-7H-pyrazolo[3,4-d][1,2,3]triazine-2-oxide, PTO, and 4-amino-7-nitro-5H-pyrazolo [4,3-d][1,2,3]triazine-2-oxide). Shreeve et al. described two long-chain nitrogen isomers, 1,2-bis(4-nitro-1H-1,2,3-triazol-1-yl) diazene (N8L) and 1,2-bis(4-nitro-1H-1,2,3triazol-2-yl)diazene (N8B) [26]. Although their energetic performances were virtually the same, their thermal stabilities differed. As summarized in Table 1, isomerization significantly affects the energy level and safety of energetic material, such as density, insensitivity, detonation velocity, and detonation pressure. These examples demonstrate that isomer design can enhance stability while retaining useful energetic performance. Collectively, these studies reveal the pivotal role of positional isomerism in governing material properties, thereby establishing it as an important design principle for advanced energetic materials [27,28,29]. However, compared with other molecular design strategies, such as energetic group engineering and molecular framework modification, systematic investigations of positional isomerism remain relatively limited, and the underlying structure–property relationships are still not fully understood. Its application to fused N-oxide frameworks—particularly pyrazolo-triazine cores—remains largely unexplored [30,31].

Our group previously reported 4-amino-7-methyl-5-nitro-7H-pyrazolo[3,4-d][1,2,3]triazine-2-oxide (MPTO), a heat-resistant energetic material with amino and nitro groups located on two fused rings [23]. In this structure, the methyl group is attached to the N7 position of the fused ring, while the amino group is located at the C4 position. Although MPTO exhibits satisfactory thermal stability and detonation performance, its mechanical sensitivity (impact sensitivity: 18 J) leaves room for improvement, and the structure–property relationships governing its behavior have yet to be fully elucidated. Building upon this concept, we address this question by designing and synthesizing positional isomers based on a pyrazolo-triazine N-oxide framework. Unlike previously reported isomeric energetic systems involving changes in molecular skeletons or key energetic groups, the present isomers possess the same fused-ring backbone and identical energetic functional groups, with only the position of the methyl substituent being altered. This molecular design enables the intrinsic influence of positional arrangement on crystal packing, intermolecular interactions, and energetic properties to be systematically investigated.

To test this hypothesis, we designed and synthesized 4-methylamino-5-nitro-7H-pyrazolo [3,4-d][1,2,3]-triazine-2-oxide (**1**), a precise isomer of MPTO in which the methyl group is repositioned to the amino side chain. Herein, we report the synthesis, comprehensive structural characterization (including single-crystal X-ray diffraction), and systematic evaluation of thermal stability, density, detonation performance, and mechanical sensitivity. Through comparative analysis of the two isomers, we establish clear correlations among substituent position, hydrogen-bonding network, crystal packing, and macroscopic properties. Our findings demonstrate that judicious isomer design can effectively decouple the energy–safety trade-off, providing a practical strategy for the development of next-generation high-performance, low-sensitivity energetic materials.

## 2. Results and Discussion

### 2.1. Synthesis

**Scheme 1:**
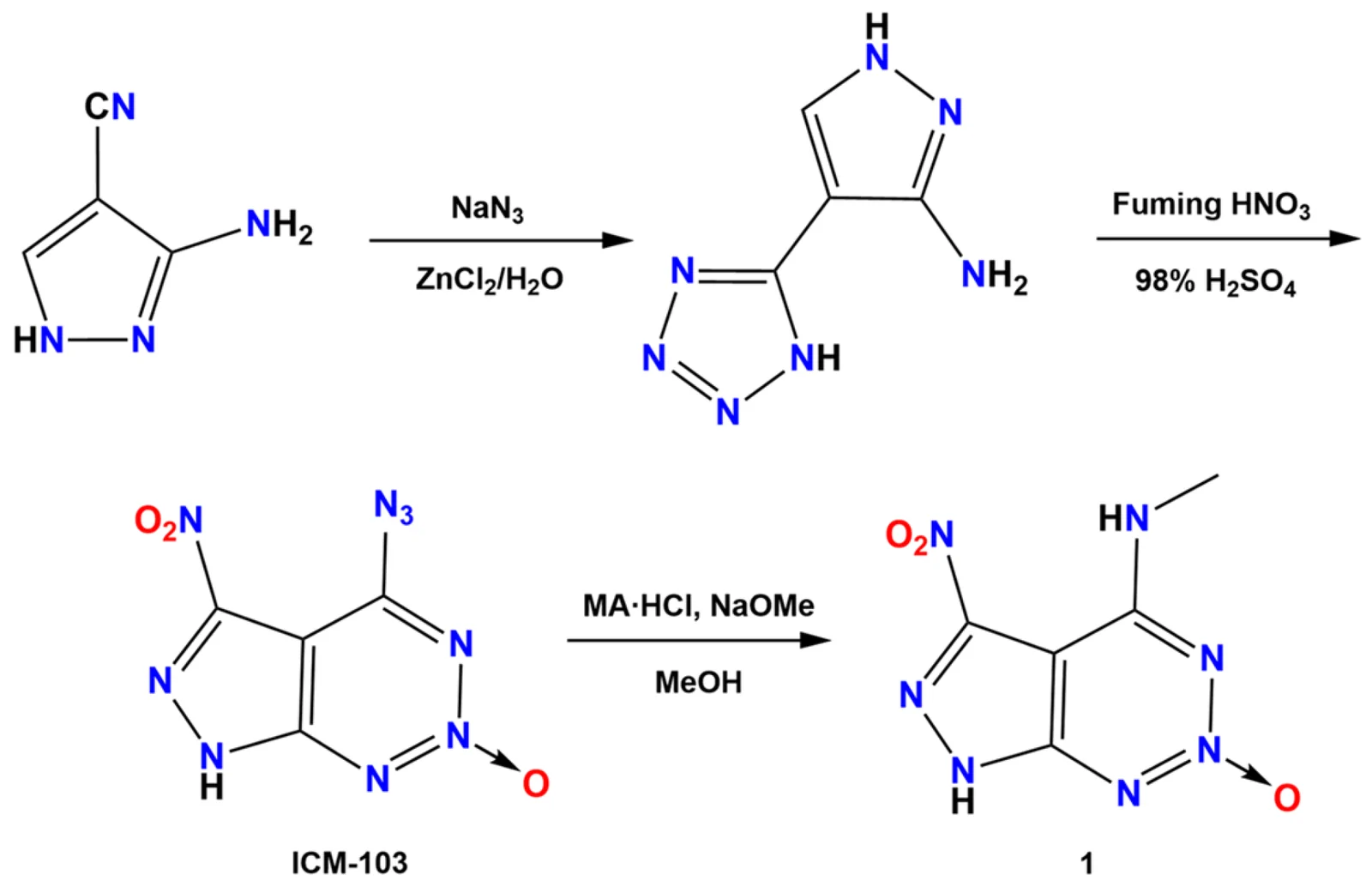
Synthesis of compound **1**.

Compound **1** was synthesized from ICM-103 via a nucleophilic substitution reaction (Scheme 1). Compound **1** was synthesized through a three-step reaction sequence employing 3-amino-4-cyan-pyrazole as the starting material. ICM-103 reacted with the newly synthesized methylamine in a methanol system at 65 °C, with the azido group being replaced by a methylamino group. The structure and purity of compound **1** were verified by NMR, IR, mass spectrometry, and elemental analysis, while its molecular structure was further confirmed by single-crystal X-ray diffraction.

### 2.2. Crystal Structure Analysis

**Figure 2:**
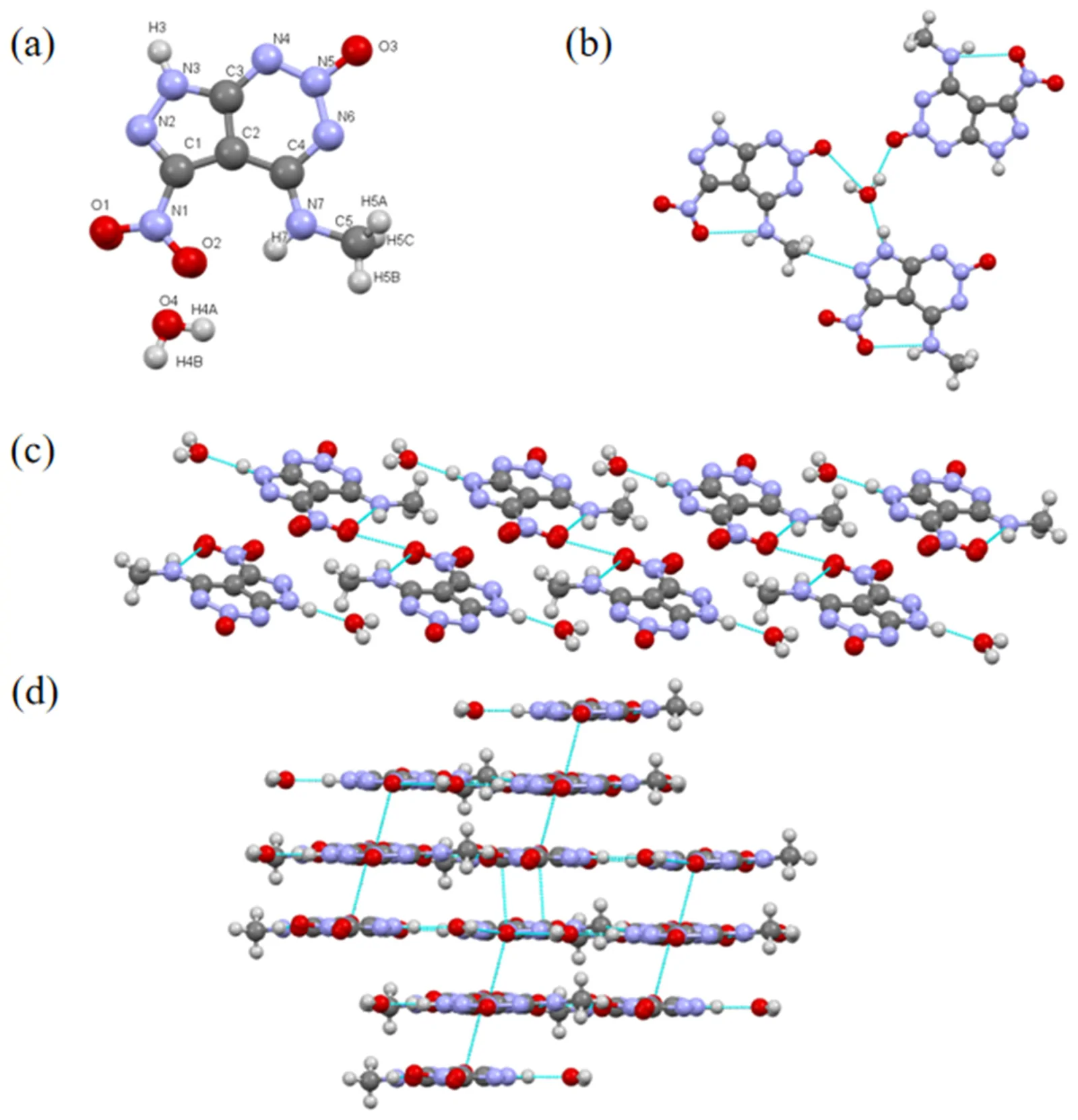
(**a**) Molecular structure of **1**·H_2_O; (**b**) hydrogen bonds formed by water molecules; (**c**) hydrogen bonds between neighboring molecules of **1**; (**d**) crystal packing. The atoms are represented by different colored spheres: gray for carbon, blue for nitrogen, red for oxygen, and white for hydrogen. The cyan dashed lines indicate intermolecular interactions.

Single-crystal X-ray diffraction analysis was carried out to gain deeper insight into the molecular structures and intermolecular interactions of the synthesized energetic derivatives. Detailed crystallographic parameters and corresponding CCDC numbers are provided in the Supplementary Materials. Crystals suitable for X-ray analysis were obtained from methanol solutions. As shown in Figure 2a, compound **1**·H_2_O crystallizes in the triclinic space group P-1, with a calculated density of 1.713 g·cm^−3^ at 100 K. The unit-cell parameters are a = 6.8314(4) Å, b = 8.3870(4) Å, c = 8.5657(4) Å, α = 72.917(2)°, β = 84.267(2)°, and γ = 71.292(2)°. The asymmetric unit comprises one molecule of compound **1** and one water molecule. Notably, all non-hydrogen atoms adopt an almost coplanar arrangement. Furthermore, an intramolecular N–H···O hydrogen bond, with a bond length of 2.840 Å, is observed, which is likely to enhance the overall molecular stability. Intermolecular hydrogen bonding between compound **1** and water gave rise to infinitely extended ribbons, three molecules in width (Figure 2b). The molecules further stack into layers (Figure 2c,d), a packing pattern that can help disperse external force and reduce sensitivity. The highly planar molecular structure favors close packing within the crystal, leading to enhanced density and consequently superior detonation performance.

### 2.3. Physicochemical and Energetic Performances

To make the experimental results more accurate, compound **1** was dried at 80 °C under reduced pressure for 8 h. The density, detonation performance, and sensitivity measurements were performed using the dried pure compound. Thermal stability is a crucial factor determining the safe manipulation and potential applications of energetic materials. The thermal decomposition characteristics of compound **1** were evaluated by simultaneous thermogravimetric analysis and differential scanning calorimetry (TG-DSC). As shown in Figure S7, compound **1** exhibits an onset decomposition temperature of 275 °C. The curve showed a melting event immediately followed by exothermic decomposition. From the DSC curve, it can be observed that compound **1** exhibited an endothermic melting event prior to exothermic decomposition, and the melting process was continuous with the subsequent decomposition. To explain this phenomenon, we conducted tests on the melting point. The melting points were measured on an MP90 Melting Point system at a heat rate of 10 °C/min. The test results showed that the melting point was 271 °C, indicating that compound **1** melted at this temperature, absorbing a substantial amount of heat and thereby generating an endothermic peak. Immediately after melting, exothermic decomposition occurred, and the peak temperature of the thermal decomposition curve of compound **1** was 296 °C, indicating that compound **1** had potential as a heat-resistant energetic material.

As a fundamental physical property, density plays a crucial role in energetic materials, governing not only their detonation performance but also their safety profile, storage stability, and practical utility. After drying at 80 °C under reduced pressure for 8 h, compound **1** showed a measured density of 1.75 g·cm^−3^. This value is comparable to those of common heat-resistant energetic materials and is slightly higher than that of MPTO.

To assess both performance and handling safety, the detonation properties of compound **1** were calculated and its mechanical sensitivities were measured. The calculated heat-of-formation values of compound **1** and MPTO were identical (340.2 kJ mol^−1^) [32], indicating that the two isomers possess comparable intrinsic chemical energy. This result is reasonable considering that both compounds share the same molecular formula and an identical pyrazolo-triazine N-oxide skeleton. Based on this value and the measured density, EXPLO5 V6.02 gave a detonation velocity of 8193 m·s^−1^ and a detonation pressure of 25.6 GPa [33]. The EXPLO5-predicted detonation parameters suggest that compound **1** possesses comparable/slightly improved theoretical energetic performance relative to MPTO. The differences in their calculated detonation parameters and experimentally measured sensitivities mainly originate from variations in molecular packing, density, and intermolecular interactions rather than differences in heat release capability. BAM tests gave an impact sensitivity of >20 J and a friction sensitivity of >360 N. These results indicate that compound **1** combines useful energy output with low mechanical sensitivity.

Table 2 compares compound **1** with MPTO and representative heat-resistant explosives. Compound **1** had a density close to that of HNS, but it possesses higher detonation velocity, higher detonation pressure, and lower impact sensitivity. Compared with PYX, compound **1** also showed better detonation performance and lower impact sensitivity, although its decomposition temperature was lower. Compared with MPTO, compound **1** demonstrated good overall performance: its theoretical detonation velocity and pressure were better, while its mechanical sensitivity was reduced. The low sensitivity is likely related to its planar molecular core, dense hydrogen-bond network, and layered crystal packing. Although compound **1** exhibited a slightly lower thermal decomposition temperature, its low synthetic cost and straightforward preparation process underscore its potential advantages for large-scale industrial production.

### 2.4. Theoretical Calculations and Analysis

**Figure 3:**
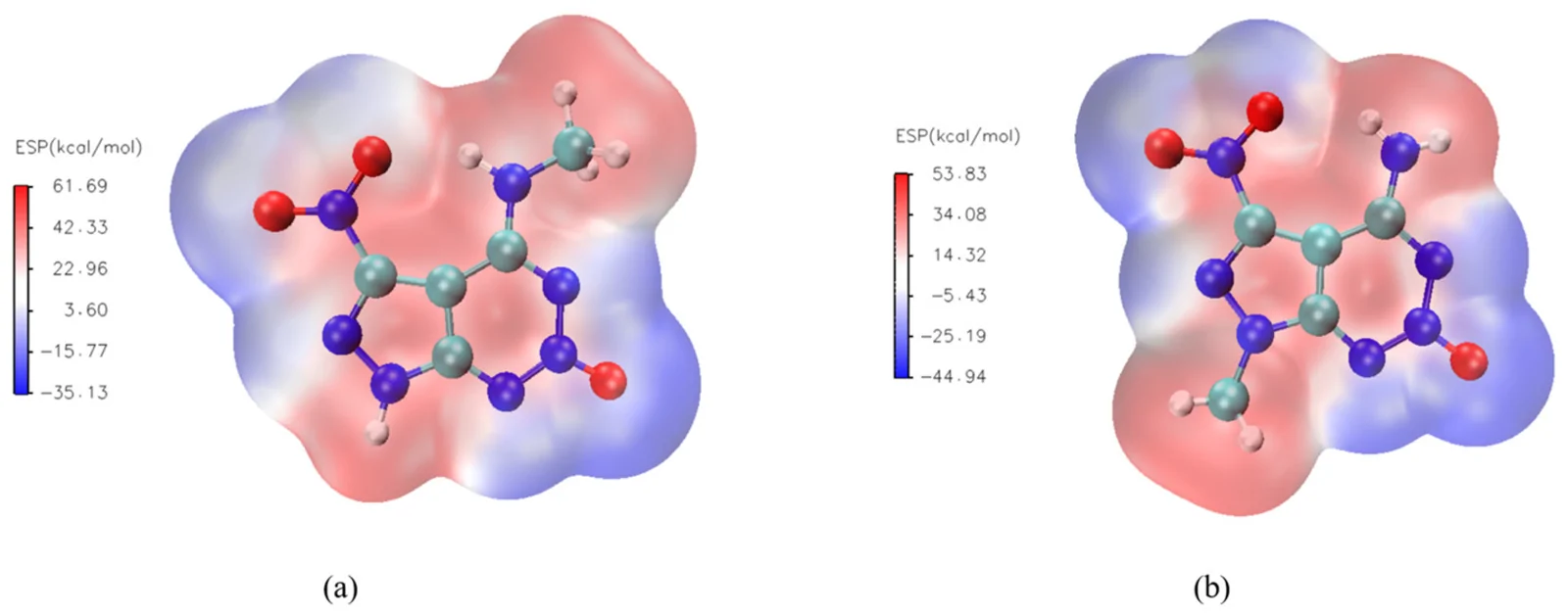
Electrostatic potential maps of (**a**) compound **1** and (**b**) MPTO. The atoms are represented by different colored spheres: gray for carbon, blue for nitrogen, red for oxygen, and white for hydrogen.

As an important indicator of safety performance, sensitivity plays a vital role in the evaluation of energetic materials. To connect molecular structure with mechanical sensitivity, electrostatic potential maps were calculated for solvent-free compound **1** and MPTO. Geometry optimization and frequency calculations were performed with Gaussian 09 at the B3LYP/6-311+G** level, and no imaginary frequencies were found. The maps were generated with Multiwfn 3.8 and Visual Molecular Dynamics (VMD 1.9.3) [34,35] (Figure 3). Negative regions are mainly located around the nitro and N-oxide oxygen atoms, whereas positive regions are found near the N-H sites. This charge pattern identifies the main hydrogen-bond acceptors and donors. For compound **1**, the distribution favors intermolecular hydrogen bonding, which helps explain its low sensitivity [36].

**Figure 4:**
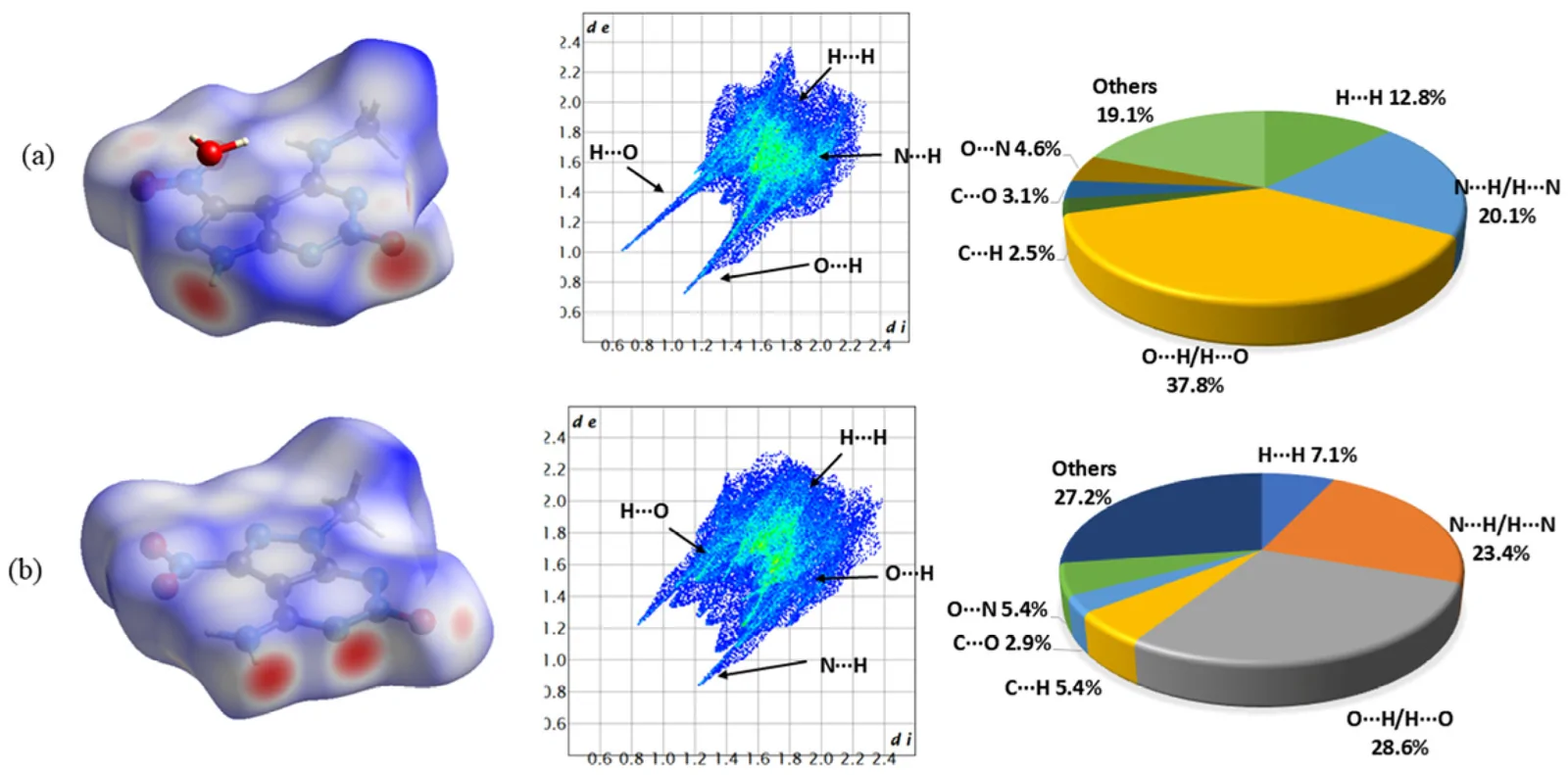
Hirshfeld surface, two-dimensional fingerprint plot, and contact contributions of (**a**) compound **1** and (**b**) MPTO.

To investigate the structural origin of the low sensitivity observed for compound **1** (IS > 20 J, FS > 360 N), Hirshfeld surface analysis and 2D fingerprint plots were utilized. Hirshfeld surface analysis was used to visualize the molecular contacts in the crystal of **1** [37]. As shown in Figure 4a, the surface was nearly plate-like, which was consistent with the planar fused-ring core. The red regions near the molecular edges correspond to short hydrogen-bond contacts. The white and blue regions on the molecular faces indicate face-to-face ring contacts and other weak contacts. The 2D fingerprint plots clearly illustrate the contributions of intermolecular interactions. The fingerprint plot showed sharp features for O···H/H···O contacts, in agreement with the hydrogen bonds observed in the crystal structure. The main contact contributions were O···H/H···O (37.8%), N···H/H···N (20.1%), H···H (12.8%), O···N (4.6%), C···O (3.1%), and C···H (2.5%). Compared with MPTO, compound **1** exhibited a higher contribution from O···H/H···O contacts (37.8% vs. 28.6%), suggesting enhanced hydrogen-bonding interactions involving oxygen atoms. These strengthened intermolecular interactions contribute to improved crystal cohesion and may inhibit the propagation of mechanical stimuli, thereby accounting for the lower sensitivity of compound **1**. The Hirshfeld surface analysis was mainly employed to elucidate the differences in intermolecular interactions between compound **1** and MPTO, rather than as direct evidence of the absolute packing characteristics of the anhydrous solid. 

**Figure 5:**
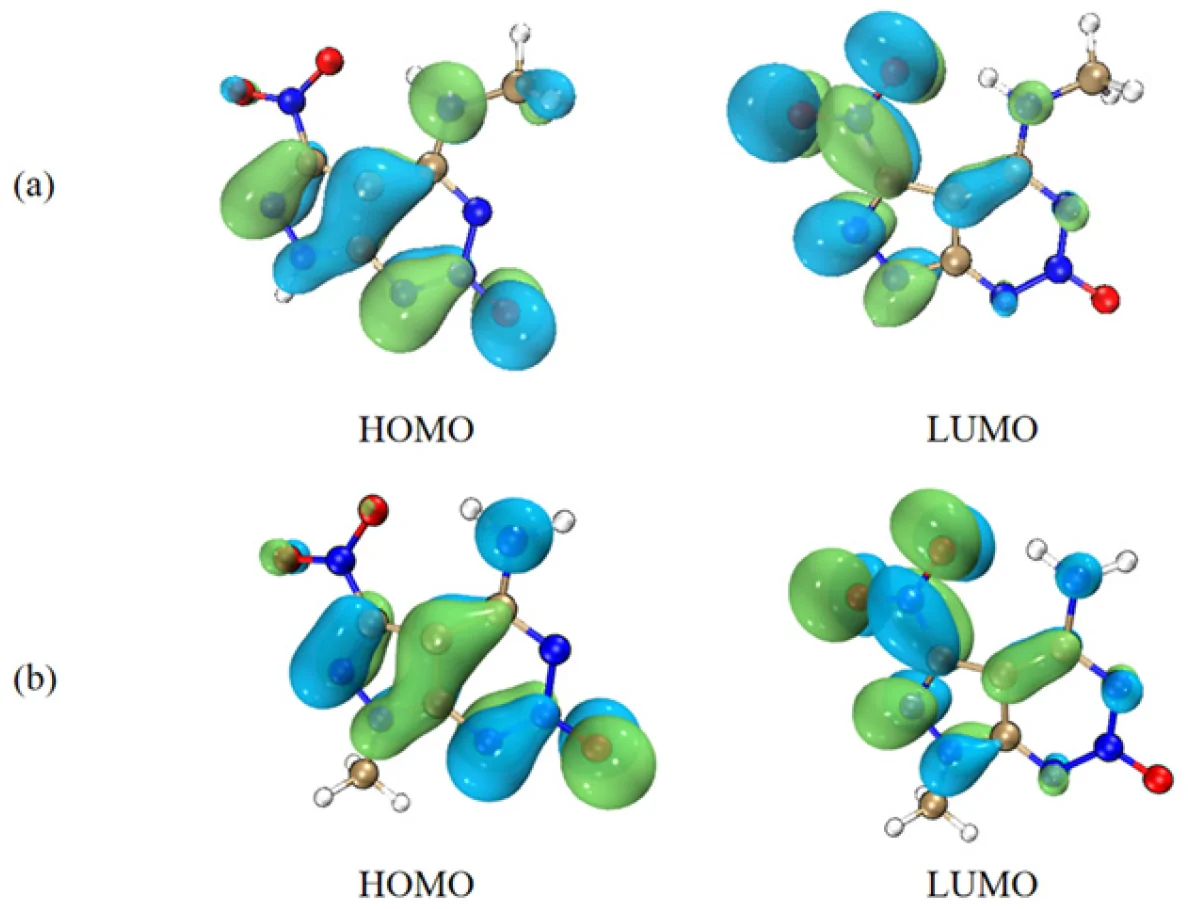
Frontier molecular orbital distributions of (**a**) compound **1** and (**b**) MPTO. The blue and green surfaces represent opposite phases of the molecular orbital wavefunctions.

To further understand the electronic structures of MPTO and compound **1**, frontier molecular orbital analyses were performed. As shown in Figure 5, the HOMOs of both compounds are mainly distributed over the nitrogen-rich fused-ring skeleton, whereas the LUMOs are primarily located around the N-oxide moiety. The similar orbital distribution indicates that the positional modification of the methyl group does not alter the fundamental electronic characteristics of the energetic framework. However, subtle differences in orbital delocalization and energy gaps suggest that positional isomerism can fine-tune the electronic stability of the molecules. As shown in Table 3, MPTO exhibits a larger HOMO–LUMO energy gap than compound **1**, which indicates enhanced electronic stability compared with compound **1**, which may partially contribute to its superior thermal stability.

It should be noted that the single-crystal structure of compound **1** was obtained in the hydrate form (**1**·H_2_O), whereas the density, energetic performance, and sensitivity measurements were performed using the dried sample. Therefore, the crystal structure of **1**·H_2_O should be interpreted with caution when correlating the molecular packing features with the bulk properties of the anhydrous compound. Nevertheless, the hydrate structure provides valuable information regarding the intrinsic intermolecular interaction patterns and molecular arrangement tendencies of this framework.

## 3. Materials and Methods

### 3.1. Reagents and Instrumentation

3-Amino-4-cyanopyrazole was purchased from Sarn Chemical Technology Co., Ltd. (Shanghai, China). Sodium azide was purchased from Sinopharm Chemical Reagent Co., Ltd. (Shanghai, China). Dimethylamine hydrochloride was purchased from Shanghai Meryer Chemical Technology Co., Ltd. (Shanghai, China). Methylamine hydrochloride was purchased from Shanghai Macklin Biochemical Co., Ltd. (Shanghai, China). N,N-Dimethylformamide was purchased from Guangdong Guanghua Sci-Tech Co., Ltd. (Shantou, China). Fuming nitric acid (95%) and methanol were purchased from Chengdu Kelong Chemical Co., Ltd. (Chengdu, China). Concentrated sulfuric acid (98%) and hydrochloric acid were purchased from Chongqing Chuandong Chemical Co., Ltd. (Chongqing, China). Sodium methoxide was purchased from Shanghai Aladdin Biochemical Technology Co., Ltd. (Shanghai, China). All reagents were used without further purification unless otherwise noted.

^1^H and ^13^C NMR spectra were recorded on a Bruker AV II-400 spectrometer (Bruker BioSpin, Ettlingen, Germany) (400 and 100 MHz, respectively) with an internal standard. The infrared (IR) spectra were recorded by a Spectrum II spectrometer using KBr pellets (PerkinElmer, Waltham, MA, USA). High-resolution mass spectra (HRMS) were obtained with SCIEX X500R QTOF MS (ESI) (SCIEX, Framingham, MA, USA). Elemental analyses (C, H, N) were performed on a Vario Micro cube elemental analyzer (Elementar Analysensysteme, Langenselbold, Germany). Powder X-ray diffraction (PXRD) patterns were collected on a Bruker AXS D8 Advance diffractometer (Bruker AXS, Karlsruhe, Germany). In situ variable-temperature PXRD measurements were taken. Single-crystal X-ray diffraction tests were performed at room temperature on a Bruker D8 Venture X-ray single-crystal diffractometer (Bruker AXS, Karlsruhe, Germany). The densities were measured at ambient temperatures by employing a gas pycnometer (Micromeritics AccuPyc II 1340) (Micromeritics Instrument Corp., Norcross, GA, USA). Thermal property data were collected using a TGA 2(SF) Mettler Toledo calorimeter (Mettler-Toledo, Greifensee, Switzerland). Impact and friction sensitivities were measured using a BFH-12 BAM impact sensitivity tester (OZM, Prague, Czech Republic) and an FSKM-10 BAM friction sensitivity tester (OZM, Prague, Czech Republic), respectively.

### 3.2. Synthesis of Compound ***1***

Compound **1** was synthesized from ICM-103 via a nucleophilic substitution reaction (Scheme 1). ICM-103 was prepared following a previously reported procedure [22]. Methylamine hydrochloride (1.36 g, 20 mmol) was suspended in methanol (7 mL), and a sodium methoxide solution (4 mL, 20 mmol) was added dropwise. The resulting mixture was stirred at room temperature for 15 min and then filtered. The filtrate was added dropwise to a solution of ICM-103 (0.28 g, 1.25 mmol) in methanol (15 mL). The reaction mixture was heated to 65 °C and stirred for 4 h. After cooling to room temperature, the precipitated solid was collected by filtration, washed with methanol, and subsequently dissolved in water. The aqueous solution was acidified to pH 2 with 10% HCl. The resulting solid was filtered, washed with water, and dried to afford compound **1** as a yellow solid. The crude product has been recrystallized by heating in a methanol solution (0.21 g, 79.6% yield). ^1^H NMR (400 MHz, DMSO-d_6_, 25 °C) δ: 7.99 (s, 1H), 3.02 (s, 3H). ^13^C NMR (101 MHz, DMSO-d_6_, 25 °C) δ: 155.03, 153.94, 148.20, 86.09, 28.06. FT-IR (KBr, ν/cm^−1^): 3512, 3427, 3373, 1584, 1530, 1453, 1382, 1293, 1177, 1091, 986, 862, 829, 747, 638, 578. HRMS (ESI^−^): calcd for C_5_H_4_N_7_O_3_^−^: 210.0381; found: 210.0373. Elemental Analysis calcd (%) for C5H_5_N7O3: C 28.44, H 2.39, N 46.44. Found: C 28.33, H 2.42, N 46.39.

## 4. Conclusions

In conclusion, compound **1** (4-methylamino-5-nitro-7H-pyrazolo[3,4-d][1,2,3]-triazine-2-oxide) was successfully synthesized from ICM-103 via a straightforward substitution reaction in 79.6% yield. As a designed isomer of MPTO, it served as an ideal model for elucidating how the positional arrangement of substituents influences the energetic properties of fused N-oxide frameworks. The structure of **1** was unequivocally confirmed by NMR, FT-IR, and single-crystal X-ray diffraction. Slow evaporation of a methanol solution afforded single crystals of the hydrate **1**·H2O, which crystallizes in the triclinic space group P-1 and features an extensive hydrogen-bonding network along with a characteristic layered packing motif. These structural attributes were recognized as favorable factors for reducing mechanical sensitivity. Compound **1** exhibits a measured density of 1.75 g·cm−3, a decomposition peak temperature of 296 °C, a calculated detonation velocity of 8193 m·s−1, and a calculated detonation pressure of 25.6 GPa. Its impact sensitivity exceeds 20 J, and its friction sensitivity exceeds 360 N. Compared with MPTO, compound **1** achieves an improved balance between energy output and safety characteristics, exhibiting comparable calculated detonation performance with reduced mechanical sensitivity, while its lower thermal stability remains an aspect requiring further optimization. These results indicate that positional modification of a substituent within an unchanged energetic framework can effectively fine-tune the balance between energy and safety in this molecular system. Collectively, this work demonstrates that positional isomerism within the pyrazolo-triazine N-oxide framework can serve as a fine-tuning strategy to regulate crystal packing, intermolecular interactions, and the balance between energy output and safety. Although this study is limited to one pair of positional isomers, it provides valuable insights into how subtle structural rearrangement without altering the energetic skeleton can influence the macroscopic properties of fused-ring energetic materials. Further systematic investigations involving diverse isomeric systems are required to establish more general predictive design principles.

## Figures and Tables

**Table 1:** Physicochemical and energetic properties of the listed compounds.

Compound	ρ ^a^ (g∙cm^−3^)	Δ_f_H ^b^ (kJ∙mol^−1^)	T_d_ ^c^ (°C)	D ^d^ (m∙s^−1^)	P ^e^ (GPa)	IS ^f^ (J)	FS ^j^ (N)
LLM-116	1.90	113.0	178	8240	29.4	>40	—
5-ADNP	1.87	60.0	198	8550	33.7	23	>360
NANP	1.75	199.0	157	8256	29.7	>40	—
ICM-103	1.86	744.7	160	9111	35.1	4	60
NAPTO	1.85	773.8	203	9120	35.1	18	325
N8L	1.78	877.7	177	8916	33.4	1.5	10
N8B	1.80	835.1	264	8917	33.5	2	20

^a^ Density (determined by a gas pycnometer at 25 °C); ^b^ calculated heat of formation; ^c^ decomposition peak temperature at a heating rate of 10 K∙min^−1^; ^d^ detonation velocity calculated using EXPLO5 V6.02; ^e^ detonation pressure calculated using EXPLO5 V6.02; ^f^ impact sensitivity evaluated by a standard BAM fall hammer; ^j^ friction sensitivity evaluated by a BAM friction tester.

**Table 2:** Detonation performance and sensitivities of compound **1** and selected energetic materials.

Compound	*ρ*/g∙cm^−3^	Δ_f_*H*/kJ∙mol^−1^	*T*_d_/°C	*D*/m∙s^−1^	*P*/GPa	*IS*/J	*FS*/N
**1**	1.75	340.2	296	8193	25.6	>20	>360
MPTO	1.73	340.2	348	8102	25.0	18	>360
HNS ^a^	1.75	78.2	318	7612	24.3	5	240
PYX ^a^	1.76	43.7	350	7757	25.1	10	360

^a^ Ref. [18].

**Table 3:** Detonation performance and sensitivities of compound **1**.

Compound	HOMO Energy/eV	LUMO Energy/eV	HOMO-LUMO Gap/kJ∙mol^−1^
**1**	−7.181	−3.604	345.115
MPTO	−7.208	−3.555	352.447

## Data Availability

The original contributions presented in this study are included in the article/Supplementary Materials. Further inquiries can be directed to the corresponding author.

## Supplementary Materials

The following supporting information can be downloaded at https://www.mdpi.com/article/10.3390/molecules31183203/s1. Figure S1: ^1^H NMR of compound **1** in DMSO-d_6_ operating at 400 MHz. Figure S2: ^13^C NMR of compound **1** in DMSO-d_6_ operating at 400 MHz. Figure S3: FT-IR tests of compound **1**. Figure S4: Negative ion mass spectrum of 1 (calcd for C_5_H_4_N_7_O_3_^−^: 210.0381). Figure S5: PXRD patterns of compound **1**. Figure S6: Crystal diagram of compound **1** with 50% probability thermal ellipsoids. The hydrogen atoms are standard balls for clarity. Figure S7: TG-DSC curve of compound **1**. Table S1: Crystal data and structure refinement of 1·H_2_O. Table S2: Table of bond lengths for compound **1**·H_2_O. Table S3: Table of bond angles for compound **1**·H_2_O. Table S4: Table of torsion angles for compound **1**·H_2_O. Table S5: Hydrogen-bond distances and angles in compound **1**·H_2_O. Table S6: Calculated zero-point energy (ZPE), values of the correction (H_T_), total energy (E_0_) and gas-state heats of formation (HOFs). Table S7: The gas-phase heats of formation (HOFs), sublimation enthalpies and solid-phase heat of formation (Δ_f_H_m_) for target compounds. Table S8: The bond dissociation energy (BDE) for the homolysis of the C-NO_2_ bond for compound **1** and MPTO. Table S9: Table of bond lengths for compound **1** at B2PLYP-D3/def2-TZVP level. Table S10: Table of bond angles for compound **1** at B2PLYP-D3/def2-TZVP level. Table S11: Table of bond lengths for compound **1** at M06-2X-D3/6-311+G(d,p) level. Table S12: Table of bond lengths for compound **1** at ωb97xd/6-311+G(d,p) level. Scheme S1: Isodesmic reactions for compound **1**. See refereneces cited in the Supplementary Materials [34,38,39,40,41].

## Abbreviations

The following abbreviations are used in this manuscript:

NANP	3-nitramine-4-nitropyrazole
LLM-116	4-amino-3,5-dinitro-1H-pyrazole
5-ADNP	5-amino-3,4-dinitropyrazole
ICM-103	6-nitro-7-azido-pyrazol[3,4-d][1,2,3] triazine-2-oxide
NAPTO	4-nitro-7-azido-pyrazol[3,4-d][1,2,3] triazine-2-oxide
N8L	1,2-bis(4-nitro-1H-1,2,3-triazol-1-yl)diazene
N8B	1,2-bis(4-nitro-1H-1,2,3triazol-2-yl)diazene
MPTO	4-amino-7-methyl-5-nitro-7H-pyrazolo[3,4-d][1,2,3]triazine-2-oxide
NMR	Nuclear Magnetic Resonance
FT-IR	Fourier-transform infrared spectroscopy
TG-DSC	Thermogravimetry–Differential Scanning Calorimetry
HNS	2,2′,4,4′,6,6′-Hexanitrostilbene
PYX	2,6-Bis(picrylamino)-3,5-dinitropyridine
VMD	Visual Molecular Dynamics
