# Supplementary material for: Isomerism as a Design Tool: Tuning the Energy–Safety Balance of a Fused Pyrazolo-Triazine N-Oxide Energetic Material
Source: Molecules. 2026 Sep 11;31(18):3203. doi: 10.3390/molecules31183203 (PMC13609284; doi:10.3390/molecules31183203)
Supplement: Supplementary file 1 [file molecules-31-03203-s001.zip › molecules-4454298-supplementary.pdf]

Electronic Supplementary Information

Isomerism as a Design Tool: Tuning the Energy–Safety Balance of a Fused Pyrazolo-Triazine N-Oxide Energetic Material

Haohuang Wang [1], Ziwu Cai [2], Tianyu Jiang [1], Yuteng Cao [1] and Wenquan Zhang [1,*]

[1] National Key Laboratory of Chemical Explosion Safety, Institute of Chemical Materials, China Academy of Engineering Physics, Mianyang, Sichuan 621900, China

[2] Extreme-Environment-Resistant Materials Key Laboratory of Sichuan Province, Sichuan University of Science & Engineering, Zigong, Sichuan 643000, China

*Corresponding authors. E-mail: zhangwq-cn@caep.cn (W.-Q. Zhang).

Contents

1. General Information S2

2. ^{1}H and ^{13}C NMR Spectra S3

3. FT-IR Spectra S4

4.High-Resolution Mass Spectrometry S4

5. In situ Variable-Temperature Powder X-ray Diffraction S5

6. Crystal Structure Data S6

7. TG-DSC curve S9

8. Theoretical Calculation S9

9. References S13

1. General Information

All chemicals were commercially-available, and they were used without further purification. ^{1}H and ^{13}C NMR spectra were recorded on a Bruker AV II-400 spectrometer (400 and 100 MHz, respectively) with an internal standard (^{1}H NMR: DMSO-d_6 at 2.50 ppm; ^{13}C NMR: DMSO-d_6 at 39.5 ppm; DMSO-d_6: D.99.9% + 1% TMS). The infrared (IR) spectra were recorded by a Spectrum II spectrometer using KBr pellets. High resolution mass spectra (HRMS) were obtained with SCIEX X500R QTOF MS (ESI). Elemental analyses (C, H, N) were performed on a Vario Micro cube elemental analyzer. Powder X-ray diffraction (PXRD) patterns were collected on a Bruker AXS D8 Advance diffractometer. In situ variable-temperature PXRD measurements were performed in the temperature range of 30–200 °C with a heating rate of 30 °C min^{-1}. Single crystal X-ray diffraction tests were performed at room temperature on Bruker D8 Venture X-ray Single crystal diffractometer. Densities (ρ) were determined by a Micromeritics Accupyc II 1340 gas pycnometer at room temperature. Thermal property data were collected using a TGA 2(SF) Mettler Toledo calorimeter with a heating rate of 10 ℃·min^{-1} under dry oxygen-free nitrogen atmosphere. Impact and friction sensitivities were measured using a BFH-12 BAM impact sensitivity tester and an FSKM-10 BAM friction sensitivity tester, respectively. The detonation velocity (D_v) and detonation pressure (P) were calculated using the EXPLO5 program (version 6.02). Hirshfeld surface analyses and two-dimensional (2D) fingerprint plots were generated with CrystalExplorer, while crystal structure visualizations and analyses were performed using the Olex2 and Diamond software packages**Error! Reference source not found.Error! Reference source not found.**.

2. ^{1}H and ^{13}C NMR Spectra

The NMR tests for the compound were performed using DMSO as solvent. The chemical shifts in the ^{1}H NMR were calibrated referring to the DMSO peak at 2.50 ppm and the chemical shifts in the ^{13}C NMR were referenced to the DMSO peak at 39.52 ppm.

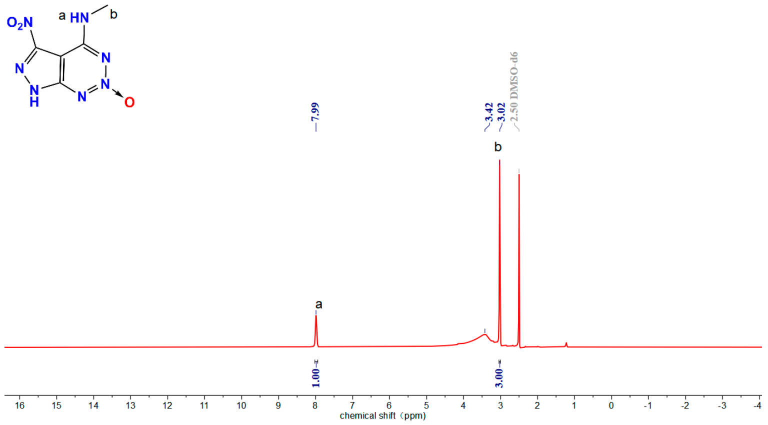

Figure S1. ^{1}H NMR of compound **1** in DMSO-d_6 at 400 MHz

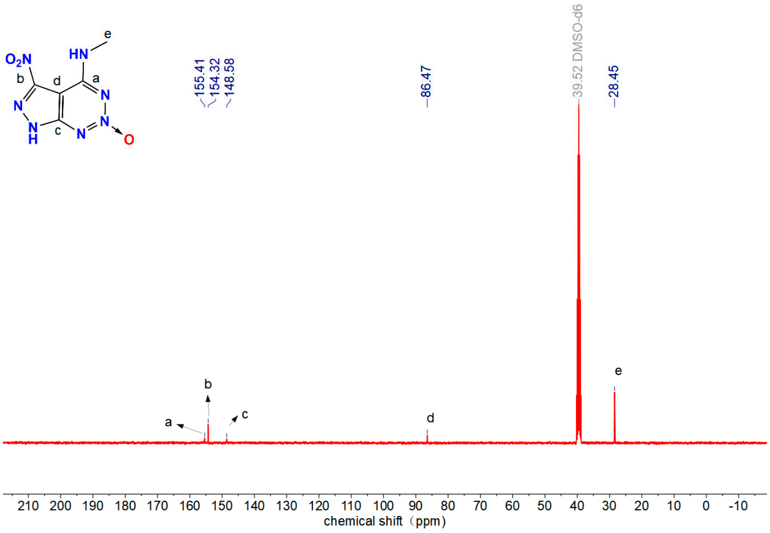

Figure S2. ^{13}C NMR of compound **1** in DMSO-d_6 at 100 MHz

3. FT-IR Spectra

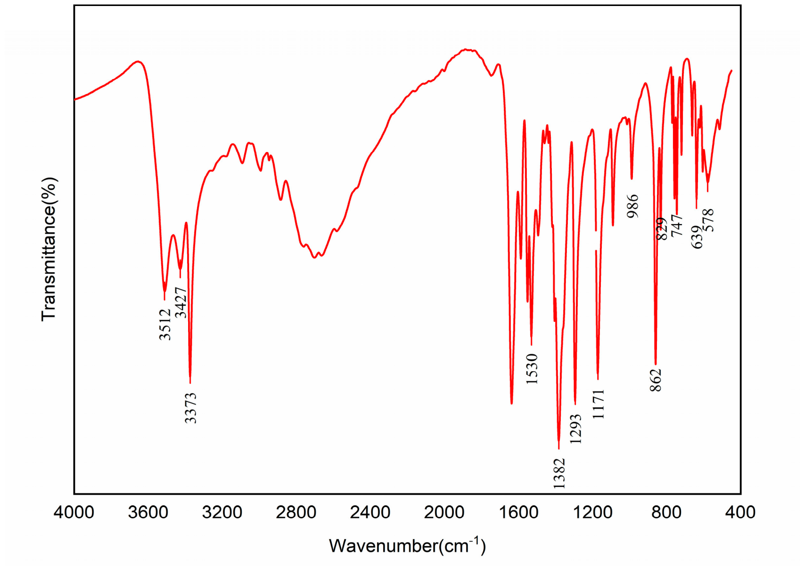

Figure S3. FT-IR spectra of compound **1**

FT-IR (KBr, cm^{-1}): 3512, 3427, 3373 (ν(N–H)), 1584 (ν(C=N)), 1530 (νas(NO2)), 1453, 1382 (ν_s(NO$_2$)), 1293, 1177 (ν(C–N)/ν(N–O)), 1091, 986, 862, 829, 747, 638, 578(ring deformation vibrations).

4.High-Resolution Mass Spectrometry

The solvent used in HRMS is dimethyl sulfoxide.

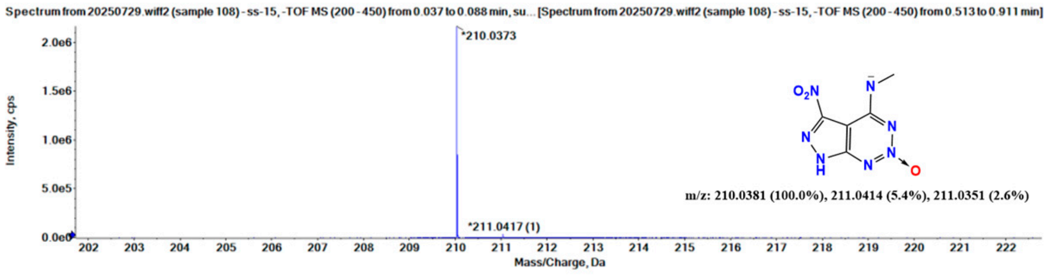

Figure S4. Negative ion mass spectrum of **1** (calcd for $C_5H_4N_7O_3^-$: 210.0381)

5. In situ Variable-Temperature Powder X-ray Diffraction

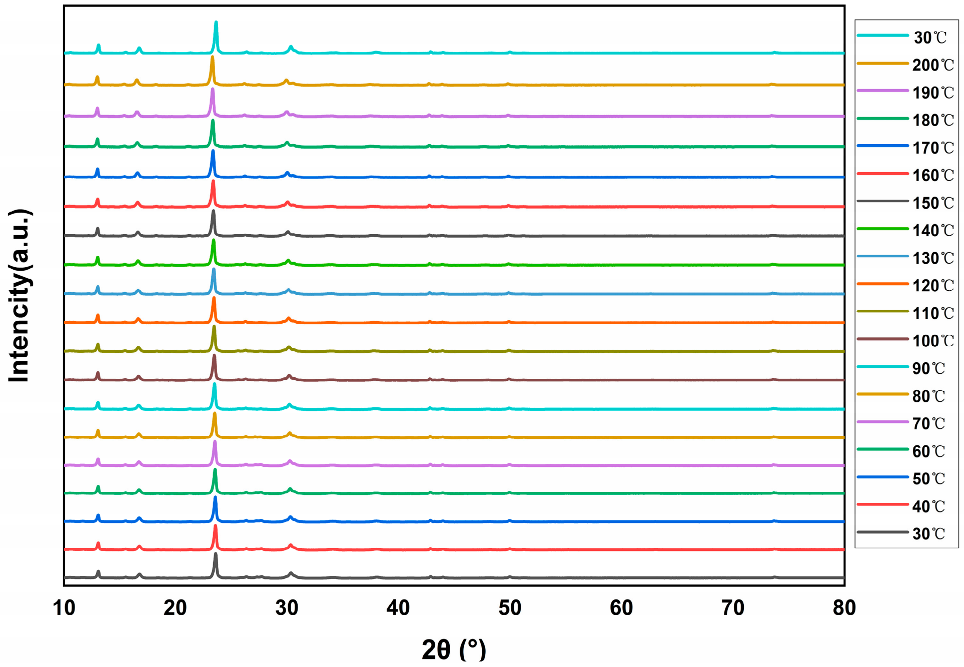

Figure S5. PXRD patterns of compound **1**

The PXRD patterns were collected to investigate the phase evolution and structural stability of the material upon heating.

6. Crystal Structure Data

Compound **1** has been recrystallized in methanol solution. The specific method involves heating it in methanol to 60 °C until dissolved, followed by cooling to induce crystallization.

The crystal structure was solved and refined by SHELXS and SHELXL-97. All non-hydrogen atoms were located from the single crystal X-ray diffraction data. Crystallographic details of the structure refinement of **1** in this work are given in Table S1-S3. The atomic coordinates and equivalent isotropic displacement parameters can be found in the corresponding CIF files.

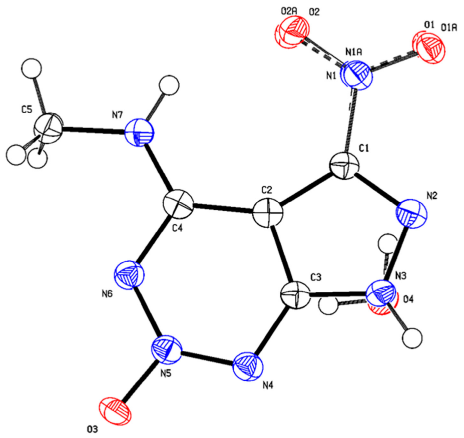

Figure S6. Crystal diagram of compound **1** with 50% probability thermal ellipsoids. The hydrogen atoms were standard ball for clarity

Table S1. Crystal data and structure refinement of **1**·H_2O

formula	$C_5H_7N_7O_4$
CCDC	2492273
Formula weight	229.18
Temperature/K	100
Crystal system	Triclinic
Space group	P-1
a/Å	6.8314(4)
b/Å	8.3870(4)
c/Å	8.5657(4)
α/(°)	72.917(2)
β/(°)	84.267(2)
γ/(°)	71.292(2)

V/ Å3	444.32(4)
Z	2
ρcalc/g·cm^{-3}	1.713
μ/mm^{-1}	0.148
F(000)	236.0
Data/restraints/parameters	1807/73/159
Goodness-of-fit on F^2	1.053
R_1,wR_2(I>=2σ (I))	R_1 = 0.0434, wR_2 = 0.1030
R_1,wR_2(all data)	R_1 = 0.0608, wR_2 = 0.1162

Table S2. Table of bond lengths for compound **1**·H_2O

bond	bond length/Å	bond	bond length/Å
O1—N1	1.220	N6—N5	1.345
O3—N5	1.269	N6—C4	1.359
O2—N1	1.237	N2—C1	1.320
N7—C4	1.313	C3—C2	1.387
N7—C5	1.460	C4—C2	1.428
N1—C1	1.444	C1—C2	1.410
N4—N5	1.313	C1—N1A	1.444
N4—C3	1.356	O1A—N1A	1.220
N3—N2	1.344	O2A—N1A	1.237
N3—C3	1.348		

Table S3. Table of bond angles for compound **1**·H_2O

Atom–atom–atom	Bond angle/º	Atom–atom–atom	Bond angle/º
H4A—O4—H4B	100.9	O3—N5—N4	117.21(15)
C4—N7—H7	118.4	O3—N5—N6	113.61(15)
C4—N7—C5	123.13(16)	N4—N5—N6	129.18(16)
C5—N7—H7	118.4	C1—N2—N3	104.90(15)
O1—N1—O2	124.1(11)	N4—C3—C2	127.54(18)
O1—N1—C1	118.7(9)	N3—C3—N4	124.19(16)
O2—N1—C1	117.0(9)	N3—C3—C2	108.28(16)
N5—N4—C3	111.17(15)	N7—C4—N6	116.64(17)
N2—N3—H3	124.2	N7—C4—C2	125.26(17)
N2—N3—C3	111.51(15)	N6—C4—C2	118.10(17)
C3—N3—H3	124.2	N2—C1—N1	117.2(6)
N5—N6—C4	118.63(16)	N2—C1—C2	113.00(16)

N2—C1—N1A	119.5(11)	C1—C2—C4	142.35(17)
C2—C1—N1	129.8(6)	N7—C5—H5A	109.5
C2—C1—N1A	127.5(11)	N7—C5—H5B	109.5
C3—C2—C4	115.30(17)	N7—C5—H5C	109.5
C3—C2—C1	102.32(16)	O1A—N1A—C1	118(2)
H5A—C5—H5B	109.5	O1A—N1A—O2A	124(2)
H5A—C5—H5C	109.5	O2A—N1A—C1	117(2)
H5B—C5—H5C	109.5		

Table S4. Table of torsion angles for compound **1**·H_2O

Atom–atom–atom–atom	Torsion angle/º	Atom–atom–atom–atom	Torsion angle/º
O1–N1–C1–N2	1(4)	N5–N6–C4–N7	179.11(15)
O1–N1–C1–C2	179(2)	N5–N6–C4–C2	−1.1(3)
O2–N1–C1–N2	−174(3)	N2–N3–C3–N4	−179.78(16)
O2–N1–C1–C2	4(5)	N2–N3–C3–C2	0.0(2)
N7–C4–C2–C3	−177.74(17)	N2–C1–C2–C3	0.0(2)
N7–C4–C2–C1	−0.2(4)	N2–C1–C2–C4	−177.7(2)
N1–C1–C2–C3	−178(2)	N2–C1–N1A–O1A	7(9)
N1–C1–C2–C4	4(2)	N2–C1–N1A–O2A	178(5)
N4–C3–C2–C4	−1.8(3)	C3–N4–N5–O3	−178.25(14)
N4–C3–C2–C1	179.77(17)	C3–N4–N5–N6	2.4(3)
N3–N2–C1–N1	178(2)	C3–N3–N2–C1	0.0(2)
N3–N2–C1–C2	0.0(2)	C4–N6–N5–O3	179.02(15)
N3–N2–C1–N1A	179(4)	C4–N6–N5–N4	−1.6(3)
N3–C3–C2–C4	178.47(15)	C2–C1–N1A–O1A	−174(4)
N3–C3–C2–C1	−0.01(19)	C2–C1–N1A–O2A	−3(10)
N6–C4–C2–C3	2.5(3)	C5–N7–C4–N6	−4.4(3)
N6–C4–C2–C1	−179.9(2)	C5–N7–C4–C2	175.86(17)
N5–N4–C3–N3	179.19(16)	N1A–C1–C2–C3	−179(5)
N5–N4–C3–C2	−0.6(3)	N1A–C1–C2–C4	3(5)

Table S5. Hydrogen-bond distances and angles in compound **1**·H_2O

D—H…A	d(D—H)/Å	d(H…A)/Å	d(D…A)/Å	∠(DHA)/ (°)
N3—H3…O4	0.88	1.81	2.658 (2)	162
O4—H4A…O3	0.87	2.13	2.919 (2)	150
O4—H4A…O6	0.87	2.48	3.289 (2)	154

O4—H4B…O3	0.87	1.92	2.772 (2)	164
N7—H7…O2	0.88	2.07	2.840 (5)	145
N7—H7…O2A	0.88	2.59	3.350 (2)	145
C3—H5B…O1	0.98	2.59	3.553 (18)	166
C3—H5B…O1A	0.98	2.56	3.510 (2)	164

7. TG-DSC curve

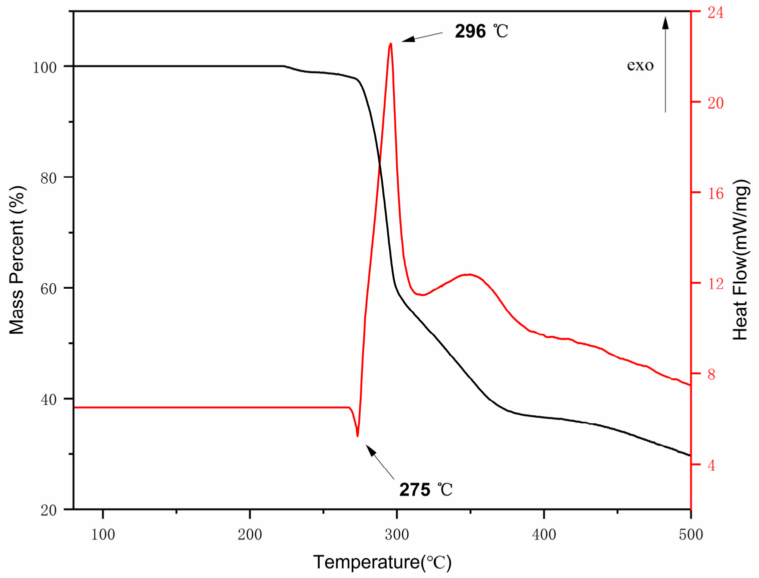

Figure S7. TG-DSC curve of compound **1**

8. Theoretical Calculation

The DFT calculations involved in this work are all performed using Gaussian 09 and Multiwfn 3.8 was used for wave function analysis**Error! Reference source not found.**. Geometry optimizations and frequency calculations were carried out at the B3LYP-D3BJ/6-311+G(d,p) level of theory. Single energy points are calculated at the B2PLYP-D3/def2-TZVP level of theory. The enthalpy of formation of compound 1 were calculated using the isodesmic reaction method.

The bond dissociation energy (BDE) for the homolysis of the C-NO_2 bond was also calculated at the B2PLYP-D3/def2-TZVP//B3LYP-D3BJ/6-311+G(d,p) according to the following equation: BDE = H(NO_2) + H(fragment) - H(whole), where H(NO_2),

H(fragment) and H(whole) are the sum of electronic and thermal enthalpies for the NO_2 radical, fragment radical and the whole geometry

For the compound, the optimized structures are characterized to be true local energy minima on the potential energy surface without imaginary frequencies. The isodesmic reaction is carried out to obtain the gas-phase heat of formation of the neutral compound (Scheme S1). The gas phase enthalpies of the building-block molecules are obtained by using the atomization method with the G4(MP2)-6x method**Error! Reference source not found.**. Then the remaining task is to determine the solid state heat of formation for the synthesized compound. The solid-state enthalpy of formation was estimated by subtracting the heat of sublimation from gas-phase heat of formation. The heat of sublimation can be estimated with Trouton's rule according to equation 1**Error! Reference source not found.**:

$$\Delta H_{sub} = 188/J{\cdot}mol^{-1}K^{-1} \times T \text{ (equation 1)}$$

Where T represents either the melting point or the decomposition temperature when no melting occurs prior to decomposition.

$$\Delta_f H(s,298K) = \Delta_f H(g,298K) \quad \Delta_{sub} H(298K) \text{ (equation 2)}$$

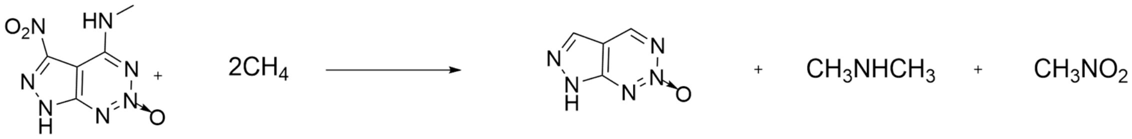

Scheme S1. Isodesmic reactions for compound **1**

Table S6. Calculated zero point energy (ZPE), values of the correction (H_T), total energy (E_0) and gas-state heats of formation (HOF)

Compound	ZPE (a.u.)	H_T (a.u.)	E_0 (a.u.)	corrected E_0 (a.u.)	HOF (kJ mol^{-1})
CH_4	0.044557	0.003813	-40.4834217	-40.4350517	-75.8
CH_3NO_2	0.049664	0.005297	-244.9481205	-244.8931595	-76.8
CH_3NHCH_3	0.092023	0.00536	-135.0850119	-134.9876289	-16.410152
	0.085901	0.007615	-502.9122588	-502.8187428	503.941022
**1**	0.133925	0.013037	-802.0217586	-801.8747966	443.2898

Table S7. the gas phase heats of formation (HOF), sublimation enthalpies and solid-phase heat of formation ($\Delta_f H_m$) for target compounds

Compound	HOF (kJ mol^{-1})	sublimation enthalpy (kJ mol^{-1})	$\Delta_f H_m$(kJ mol^{-1})
**1**	443.2898	103.0898	340.2

Table S8. The bond dissociation energy (BDE) for the homolysis of the C-NO_2 bond for compound **1** and MPTO

Compound	BDE (kJ mol^{-1})
**1**	303.39
MPTO	304.39

Coordinates of compound **1** in theoretical calculations

C -1.34620100 3.22996100 -0.00034500

N -0.39188100 2.12973200 -0.00001100

C -0.75732800 0.84706000 0.00023500

N -2.07453400 0.58968600 0.00010200

N -2.51230000 -0.69650600 0.00009500

O -3.72618100 -0.85981200 0.00001700

N -1.73339700 -1.78745500 -0.00005000

C -0.43356400 -1.49530200 -0.00002700

C 0.17255500 -0.23460200 0.00030300

C 1.56293500 -0.54069600 -0.00006900

N 2.68969700 0.37640500 0.00005500

O 3.81502400 -0.07391100 -0.00008500

O 2.40959200 1.58141100 0.00035900

N 1.79640500 -1.83692400 -0.00044800

N 0.57966900 -2.41070400 0.00001700

H -1.98556600 3.18891800 0.88353900

H -1.98605900 3.18795400 -0.88382400

H -0.78451900 4.16243900 -0.00098200

H 0.60454400 2.30785600 -0.00013600

H 0.49811800 -3.41682900 0.00017400

no imaginary frequencies.

Table S9. Table of bond lengths for compound **1** in B2PLYP-D3/def2-TZVP level

bond	bond length/Å		bond	bond length/Å
C1—H18	1.089		C8—C9	1.399
C1—H17	1.092		C9—C3	1.426
C1—H16	1.092		C9—C10	1.424
C1—N2	1.456		C10—N11	1.453
N2—H19	1.012		N11—O13	1.237
N2—C3	1.334		N11—O12	1.212
C3—N4	1.342		C10—N14	1.317
N4—N5	1.359		N14—N15	1.341
N5—O6	1.225		N15—C8	1.366
N5—N7	1.340		N15—H20	1.009
N7—C8	1.332			

Table S10. Table of bond angles for compound **1** in B2PLYP-D3/def2-TZVP level

Atom–atom–atom	Bond angle/º	Atom–atom–atom	Bond angle/º
O11—N11—O12	124.896	N7—N5—N4	125.678
O13—N11—C10	116.057	N5—N4—C3	119.852
O12—N11—C10	119.047	N4—C3—C9	119.629
N11—C10—N14	118.933	C3—C9—C10	143.101
C10—N14—N15	105.037	N4—C3—N2	116.959
N14—N15—H20	119.881	C3—N2—H19	116.038
N14—N15—C8	112.657	C3—N2—C1	123.159
N15—C8—C9	106.419	N2—C1—H16	110.804
N15—C8—N7	125.236	N2—C1—H17	110.804
C8—N7—N5	112.858	N2—C1—H18	107.999
N7—N5—O6	117.863	C1—N2—H19	120.802

Table S11. Table of bond lengths for compound **1** in M06-2X-D3/6-311+G(d,p) level

bond	bond length/Å	bond	bond length/Å
C1—H18	1.088	C8—C9	1.391

C1—H17	1.091	C9—C3	1.427
C1—H16	1.091	C9—C10	1.420
C1—N2	1.454	C10—N11	1.456
N2—H19	1.011	N11—O13	1.222
N2—C3	1.330	N11—O12	1.199
C3—N4	1.336	C10—N14	1.305
N4—N5	1.352	N14—N15	1.338
N5—O6	1.214	N15—C8	1.358
N5—N7	1.324	N15—H20	1.010
N7—C8	1.334		

Table S12. Table of bond lengths for compound **1** in ωb97xd/6-311+G(d,p) level

bond	bond length/Å	bond	bond length/Å
C1—H18	1.089	C8—C9	1.389
C1—H17	1.092	C9—C3	1.425
C1—H16	1.092	C9—C10	1.420
C1—N2	1.451	C10—N11	1.453
N2—H19	1.011	N11—O13	1.226
N2—C3	1.329	N11—O12	1.202
C3—N4	1.336	C10—N14	1.307
N4—N5	1.353	N14—N15	1.338
N5—O6	1.216	N15—C8	1.357
N5—N7	1.327	N15—H20	1.008
N7—C8	1.334		

The results revealed extremely high structural consistency, with SOAP similarity values of 99.99% for both M06-2X-D3/6-311+G(d,p) and ωb97xd/6-311+G(d,p) calculations. These results indicate that the optimized molecular structure is insensitive to the choice of functional and support the reliability of the B3LYP method employed in this study.

9. References

[S1] Frisch, M. J.; Trucks, G. W.; Schlegel, H. B.; Scuseria, G. E.; Robb, M. A.; Cheeseman, J. R.; Scalmani, G.; Barone, V.; Mennucci, B.; Petersson, G. A.; Nakatsuji, H.; et al. Gaussian 09, Revision D.01, Gaussian, Inc., Wallingford CT, **2013**.

[S2] Lu, T.; Chen, F. Multiwfn: A Multifunctional Wavefunction Analyzer. J. Comput. Chem. **2012**, 33, 580-592. https://doi.org/10.1002/jcc.22885.

[S3] Lu, T. A Comprehensive Electron Wavefunction Analysis Toolbox for Chemists, Multiwfn. J. Chem. Phys. **2024**, 161, 082503. https://doi.org/10.1063/5.0216272.

[S4] Chan, B.; Deng, J.; Radom, L. G4(MP2)-6X: A Cost-Effective Improvement to G4(MP2). J. Chem. Theory Comput. **2011**, 7, 112 – 120. https://doi.org/10.1021/ct100542x.

[S5] Westwell, M. S.; Searle, M. S.; Wales, D. J.; Williams, D. H. Empirical Correlations between Thermodynamic Properties and Intermolecular Forces. J. Am. Chem. Soc. **1995**, 117 (18), 5013 – 5015. https://doi.org/10.1021/ja00123a001.
